# Edge of the Present: A Virtual Reality Tool to Cultivate Future Thinking, Positive Mood and Wellbeing

**DOI:** 10.3390/ijerph18010140

**Published:** 2020-12-28

**Authors:** Stephanie Habak, Jill Bennett, Alex Davies, Michaela Davies, Helen Christensen, Katherine M. Boydell

**Affiliations:** 1 Black Dog Institute, School of Psychiatry, University of New South Wales, Randwick, NSW 2031, Australia; s.habak@blackdog.org.au (S.H.); h.christensen@blackdog.org.au (H.C.); 2 UNSW Art and Design, University of New South Wales, Paddington, NSW 2021, Australia; j.bennett@unsw.edu.au (J.B.); alex.davies@unsw.edu.au (A.D.); 3 Psychologist, East Sydney Doctors, Darlinghurst, NSW 2010, Australia; michaeladavies@gmail.com

**Keywords:** suicide, depression, future thinking, hopelessness, virtual reality, mixed reality, negative imagery, mood, well-being

## Abstract

Depression and suicidality are characterized by negative imagery as well as impoverished positive imagery. Although some evidence exists supporting the link between positive imagery and enhanced mood, much work needs to be done. This study explored the impact of an immersive virtual reality experience (Edge of the Present—EOTP) on an individual’s mood, state of well-being, and future thinking. Using a 10-min mixed reality experience, 79 individuals explored virtual landscapes within a purposefully built, physical room. A pre and post survey containing mental health measures were administered to each participant. An optional interview following the virtual work was also conducted. The results indicated that positive mood and well-being increased significantly post-intervention. Hopelessness scores and negative mood decreased, whilst sense of presence was very high. This pilot study is among the first to assess the feasibility of a mixed reality experience as a potential platform for depression and suicide prevention by increasing well-being and mood as well as decreasing hopelessness symptoms.

## 1. Introduction

The etiology of suicide has been researched extensively, with previous suicidal behavior being one of the strongest predictors of future suicidal attempts [1]. However, in recent years, key advances within suicide research demonstrated that a non-clinical, psychological state defined as hopelessness has been critical in understanding mental health problems. Characterized by low levels of positive future thinking, hopelessness has provided valuable insight into the psychological variables that contribute to depression [2] and suicidal behavior [3].

In the development of the personal future task (PFT), MacLeod, Rose, and Williams [4] determined that parasuicidal respondents scoring high in hopelessness, in comparison to a control group, exhibited a lack of positive future thinking, with no corresponding increase in negative future thinking. In subsequent research, MacLeod et al. [5] addressed the limitations postulated by the previous study by differentiating between depressed and non-depressed parasuicidal individuals in examining whether hopelessness or depression were significant predictors of suicidality. Their study results supported the theory that suicidality, in the absence of depression, still generated a low number of positive future events. Despite suicide acting as a major risk factor for those individuals, addressing hopelessness, when statistically controlled for, could be successful in preventing suicide [6]. 

Similarly, the ability to imagine negative or positive future events can play an important role in the process of recovery, persistence, and relapse of depression [7]. Although suicidality can exist independently of depression, Beck et al. [8] posited hopelessness as a core component of the “cognitive triad”, commonly characterized as “negative expectations about the future”. In attempting to replicate previous research [5], MacLeod and Salaminiou [2] conducted a study examining the cognitive and affective explanations for the lack of positive anticipation in depression. The results of the study deduced that, like earlier research, the decline in positive future thinking proved to be the most powerful discriminator between depressed and control participants. 

Empirical evidence also suggests that abstract/verbal processing during the recollection of autobiographical memories is also a powerful determinant in the maintenance of depressive symptomology. Autobiographical memory, the sub-system of episodic memory, which relates to personal experiences, is thought to serve various helpful functions in relation to well-being, including forming a sense of identity and growth [9]. However, the association between depression and autobiographic memory suggests that people exhibit a diminished ability to access positive memories and a tendency to recall positive events in little detail. Furthermore, personal events are recollected in an “overgeneral” way, allowing for memories to be grouped into themes and chapters, rather than recalled as individual, specific events [9]. Similarly, this concept also applies to future episodic thinking, as the same neural pathways are activated in memory and imagination. According to Slofstra et al. [10], overgeneral autobiographical memory as an abstract/verbal processing mode is characterized by weaker affective responses than imagery-based processing regarding memory retrieval. Therefore, despite these cognitions often being conceptualized as verbal, thinking in imagery (as opposed to words) can powerfully impact emotion. 

Mental imagery-based processing, or sensory processing, is proposed to enhance the impact of emotional material on negative and positive affect [10,11]. Imagery can “hijack” attention through its highly absorbing nature and sense of realness, which can be used to access autobiographical memories and their associated emotions [12]. Recently, research exploring the differential effects of verbal versus imagery-based processing determined that during the recall of positive autobiographical memories, greater levels of positive affect were achieved for the sample assigned to the sensory processing group [13]. Importantly, prospective imagery has been shown to be causal in determining future behavior; thus, imagining oneself completing a future event could lead to a significantly greater likelihood of this event being completed in real life [12]. A prospective study led by Holmes et al. [14] reported that suicidal participants at their penultimate level of crisis were likely to experience suicide-related mental images, or “flash-forwards”, of future suicidal plans or being dead. These suicidal images may be particularly toxic given the powerful effect of imagery, with its ability to hijack attention and promote behavioral action [12,15]. These findings suggest that treatment interventions targeting positive future thinking and positive affect can be addressed on two levels: through an individual’s verbal cognitions, and through mental imagery. 

The role of psychological and behavioral therapy regarding the future in depression and suicide is already reflected in common goals of cognitive behavioral therapy, including countering hopelessness and fostering a long-term perspective [16]. However, with the emergence of new technologies within the clinical sphere, virtual reality (VR) provides a powerful means for addressing the shortcomings of traditional therapies [17] regarding mental imagery and positive future thinking. According to Bouchard and Rizzo [18] in their book “Virtual Reality for Psychological and Neurocognitive Interventions”, VR can be used to create relevant simulated environments where the testing, training, teaching, and treatment of cognitive, emotional, and sensorimotor processes can take place under stimulus conditions that are not easily deliverable and controllable in the physical world. Virtual environments are, thereby, known to produce physiological changes consistent with emotional responses to real-world scenarios [19]. Moreover, using “standalone” or wireless head-mounted displays (HMDs), users are immersed in both clinical and non-clinical environments that change in a natural or intuitive way according to head and body motion, thus creating emotionally evocative therapeutic experiences. Virtual reality may also be deployed within a “mixed reality” methodology, whereby virtual elements are integrated into—and are responsive to—the natural world [20]. A mixed reality environment, thus, incorporates the senses, allowing people to interact with the virtual world using their body, with real world consequences. Virtual reality enables one to exercise agency as it is not simply a passive viewing experience but one where choice issues a pleasurable response. 

Despite the ever-growing trend to incorporate virtual reality technologies within therapeutic practices, limited research has evaluated the use of VR as a preventative or treatment measure with depressive conditions and suicidality. In 2016, Falconer et al. [21] utilized an immersive VR scenario to address self-criticism, known to be a major psychological factor inhibiting the recovery of depressed patients. Using virtual embodiment, participants practiced delivering compassion as an adult, and receiving compassion in a child-like state. The research demonstrated a significant reduction in depression severity and self-criticism, as well as increases in self-compassion after three intervention sessions. In terms of neuropsychological performance, two further studies found no differences in performance on a VR spatial navigation task [22] and a computerized cognitive training device [23] for depressed patients. However, another study measuring the performance on a VR navigation measure of visual-spatial memory found that participants with a depressive disorder performed worse than controls [24]. Furthermore, to our knowledge, only one study has applied virtual reality technology to explore suicide—specifically, the causal effects of suicide and their effects on VR suicide rates [25]. A translational approach was established to observe the effects of two VR suicide scenarios, including jumping from heights and shooting oneself, with possible predictors for engaging in suicide. The results demonstrated that suicidal desire, suicidal capability, agitation, and prior suicidality were significant predictors for engaging in VR suicide. 

Given the research into positive future thinking and its associated effect in people with depression and suicidality, our aim was to explore the relationship between hopelessness and future thinking by adopting a mental imagery-based processing model, utilizing virtual reality technology. This paper describes a pilot study, the goal of which was to establish whether an immersive, mixed reality project—Edge of the Present—could impact an individual’s mood, state of well-being, and future thinking. This immersive environment was developed for The Big Anxiety—festival of people and art and science—a Sydney-wide (and beyond) research-driven mental health festival (www.thebiganxiety.org). It was hypothesized that after experiencing the 10-min virtual experience, the mixed-reality environment yielding positive imagery would also cultivate an associated positive affect, acting as a basis for the production of positive future thinking or episodic imagining that was emotion-laden. Furthermore, through the enactment of real-life choices within the mixed reality environment, it was anticipated that a large number of positive future expectancies would directly correlate with low levels of hopelessness. With reference to overgeneral autobiographical memory, we hypothesized that engagement with Edge of the Present—and specifically the association of positive affect and sensation with actions initiated by the participants—would lead to improvements in overall mood and well-being. Finally, using a mixed reality design also allowed us to gauge the associated benefits of this new technology as a potential intervention for depressive conditions and suicide prevention. 

## 2. Materials and Methods

### 2.1. Participants

Seventy-nine participants were recruited through The Big Anxiety’s website, the Black Dog Institute’s social media platforms (Facebook, Twitter, e-newsletters) and Lived Experience Advisory Panel via an expression of interest (EOI) advertisement, as well as on-the-spot recruitment from attendees of The Big Anxiety festival. All participants were 18 years of age and older, and identified as reporting a mental health diagnosis, previous experience with suicidality, or self-identified with depression. Of this sample group, 54 participants indicated that they had previously sought professional help for their mental health challenges. Participants were excluded from the study if they reported severe mental health problems e.g., psychosis, severe mood disturbance and/or mania (with only two meeting this criterion). All participants were provided with an information sheet and consent form on the day before participating in the study. Ethics clearance was obtained by the author-affiliated university.

### 2.2. The Intervention

Edge of the Present (EOTP) is a mixed reality environment purpose designed by an artistic team led by artist Alex Davies as part of an art–science research collaboration focusing on memory and future thinking (lead researcher, Jill Bennett). It was developed in conjunction workshops with young men who experienced suicidality (facilitated by JR Brennan and partly informed by the work of Holmes et al. on suicidal imagery, which was discussed in relation to the men’s own experiences of feeling themselves to be at “the edge of the present” with no sense of a (positive) way forward). 

Edge of the Present is comprised of a sparsely furnished room with doors, windows, and a table. Upon entering the room, users don a VR headset through which they see a virtual facsimile of the room they occupy. They are invited to physically explore the room alone for 10 min, opening doors and windows freely as they choose. Edge of the Present is designed to reward such exploration—and the basic action of opening a door or window—with positively experienced imagery. Hence, the door opens onto a series of spectacular immersive landscapes (seven different vistas, including Alpine scenes, lush rainforest, tropical beaches, and a desert), accompanied by environmental effects such as a warm breeze, intensifying sensory experience. This intensification of user experience aimed to compensate for the failure to generate affect in relation to episodic memory or future thinking (c.f. Holmes’ [14] description of overgeneral autobiographical memory whereby events or episodic memories are recalled only in a general way without detail or affective association) and instead to reinforce embodied sensory-affective experience, linking this to user choice. Users find they can change the scene by opening and closing the door multiple times. Opening a window also effects change to the room itself, causing walls to disappear and the room to fill with grass, for example. 

Users are offered an actual experience in relation to moving through and acting within a mixed reality environment to enhance and intensify image generation, with the potential to bolster protective effects. The virtual experience provided users with the opportunity to be transported to seven different landscapes by interacting with a sparse, virtual room through its doors and windows (which act as portals). The greater the engagement with the room (i.e., opening and closing a door/window), the more increasingly enriched the bare room becomes by the outside landscape (i.e., ferns growing inside the room). The Edge of the Present provokes a sense of hopeful anticipation—each time the door is opened there is a new landscape for the user to experience and incorporate into their personal world (the room). Through their virtual explorations, the user learns both that openness and curiosity lead to positively reinforcing experiences, and that elements of these experiences/environments become integrated into the room they are inhabiting (i.e., enrich the internal world of the user). The user’s experience is about being present, open, and curious.

### 2.3. Design

Participant experience was evaluated using standardized measures via a pre-post methodology to identify improvements and changes in mood, future thinking, and general well-being before and after the virtual experience. Statistical analyses, including *t* tests and a mixed model ANOVA were used to examine changes over time, as well as its effect on demographic variables including age, sex, employment status, education level, virtual reality experience, and whether or not mental health help was previously sought. 

### 2.4. Procedure 

After agreeing to participate and following written consent, participants were provided an electronic tablet to complete an online survey using the Qualtrics application. Mental health and well-being assessments were completed immediately prior to the virtual experience. Following completion of the “pre” survey, participants experienced the 10-min virtual reality program. Once completed, the “post” survey was administered to participants with the option of providing open-ended comments about their experience. 

### 2.5. Outcome Measures 

Basic demographic form: Basic demographic information was collected including age, sex, spoken language, education status, employment status, experience with virtual reality, and whether prior help had been sought from a mental health professional.

Short Warwick–Edinburgh Mental Well-Being Scale (SWEMWBS): The SWEMWBS is a shortened version of the original Warwick–Edinburgh Mental Well-Being Scale. The scale was developed to enable the monitoring of mental well-being in the general population. The SWEMWBS uses 7 of 14 statements, all of which are positively worded. Responses are made on a 5-point scale ranging from “none of the time” to “all of the time” [26]. 

Beck Hopelessness Scale: The Beck Hopelessness Scale (BHS) is a 20-item self-report scale that was developed to operationalize the construct of hopelessness, assessing three major aspects: feelings about the future, loss of motivation, and future expectations [27]. Individual scale items are measured using a dichotomous, true-false response format. Each optimistic response is scored as 0 and each pessimistic response is scored as 1. A total score is derived by summing the pessimistic responses for each of the 20 items. Scores range from normal (0–3), mild hopelessness (4–8), moderate hopelessness (9–14), and severe hopelessness (>14) [27]. 

The Positive and Negative Affect Schedule (PANAS): The Positive and Negative Affect Schedule [28] is a widely used measure of mood or emotion. The scale is comprised of 20 items; 10 items measuring positive affect (e.g., enthusiastic) and 10 items measuring negative affect (e.g., hostile). Affect is measured according to a specified time frame using a 5-point Likert scale, ranging from “1 = Very slightly or not at all” to “5 = Extremely”. Thus, the scale can be used to measure state affect, dispositional or trait affect, emotional fluctuations throughout a specific period of time, or emotional responses to event [29]. 

Sense of Presence: Three questions were used to evaluate the participant’s sense of presence in the virtual environment, using a Likert scale ranging from “1 = Poor” to “5 = Excellent”. Presence is crucial for participants to feel as if they are in a virtual environment, rather than merely observing it [30]. Questions were taken from two measures [31,32]. These questions were:

1. What was your overall enjoyment level in this environment?
2. How strong was your sense of presence, “being there”, in the virtual environment?
3. What was your overall comfort level in this environment?

## 3. Results

### 3.1. Demographics

The sample was comprised of 79 participants (53 female, 23 male, and 3 non-binary), with an average age range between 25–34 years (24.1% of total sample). Two male participants aged 25 and 44 were excluded from the study for not meeting the inclusion criteria. Most participants spoke English as their first language (73.4%) and either attended university or college (87.3%). Fifty-four individuals (68.4%) had previously sought help from a mental health professional. Most participants were employed part-time (31.6%), full-time (30.4%), or were students (17.7%). Fifty-one participants (64.6%) indicated “quite a bit” of experience with virtual reality. Table 1 provides a summary of participant characteristics.

### 3.2. Hopelessness, Well-Being, and Mood

The primary outcome measure of hopelessness, as measured by the Beck Hopelessness Scale, was assessed prior to The Edge of the Present (*M* = 5.32, *SD* = 4.31) and again after the immersive virtual reality session (*M* = 3.44, *SD* = 3.12). A repeated-measures *t*-test found a statistically significant decrease in levels of hopelessness, *t* (78) *=* 5.18, *p* < 0.001 from pre to post. Changes in positive (*M* = 30.71, *SD* = 7.87) and negative mood (*M* = 16.98, *SD* = 5.75) were assessed using the Positive and Negative Affect Schedule at baseline. A significant increase in positive mood (*M* = 35.91, *SD* = 6.37) was found following the virtual experience *t* (78) *=* −8.26, *p* < 0.001, with a corresponding decrease in negative mood (*M* = 14.13, *SD* = 4.44), suggesting that 10 min within the immersive work can have a positive impact on an individual’s mood, *t* (78) *=* 6.49, *p* < 0.001. For well-being, as measured by the SWEMWBS, scores were assessed prior to the experience (*M* = 3.36, *SD* = 0.64) and once completed (*M* = 3.80, *SD* = 0.53). The results demonstrated a significant increase in well-being at post following the participant’s involvement in The Edge of the Present, *t* (78) *=* −7.21, *p* < 0.001. These results are summarized in Table 2. 

### 3.3. Mixed Model Anova

A mixed model ANOVA was used to observe the interaction between each of the demographic variables including age, sex, employment status, education level, virtual reality experience, and previous help seeking on the mental health measures before and after the virtual work. Of the variables, only “employment” and “sought help” displayed significant main effects on hopelessness and well-being scores. No interaction effects were observed regarding mood and the covariables.

#### 3.3.1. Hopelessness

**Figure 1:**
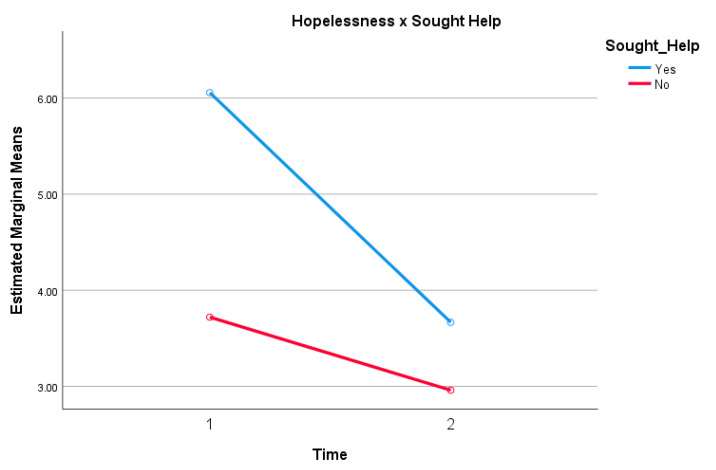
Observed interaction effect of hopelessness and previous help-seeking behavior. Sense of Presence.

A significant main effect for employment (Table 3) was reported, F (8, 70) = 3.114, *p* = 0.005, partial η^2^ = 0.262, with hopelessness scores after Edge of the Present being significantly lower than before the virtual work. Examination of the means indicated that hopelessness scores across all employment types excluding those ”not employed, not looking for work”, “self-employed”, “disabled, unable to work”, and “other”, significantly decreased after participating in the virtual work. Table 2 summarizes the results from pre to post, as well as the change scores, with the greatest reduction found amongst those “not employed, looking for work” (*M* = 2.80, *SD* = 4.15). Similarly, a significant main effect for the variable “sought help” was obtained F (1, 77) = 4.60, *p* =0.035, partial η^2^ = 0.056 (Figure 1). Examination of the means indicated that participants who previously sought help for their mental health (*M* = 6.06, *SD* = 4.51) demonstrated the greatest decline in hopelessness scores following Edge of the Present (*M* = 3.66, *SD* = 3.49). The participant group who did not seek help for their mental health also exhibited a smaller, yet significant decline in hopelessness scores at pre (*M* = 3.72, *SD* = 3.39) versus post (*M* = 2.96, *SD* = 2.11).

#### 3.3.2. Well-Being

A significant main effect for the interaction between well-being and employment was reported, F (8, 70) = 3.06, *p* = 0.005, partial η^2^ = 0.223. Examination of the means indicated that wellbeing scores across all employment types, excluding those “not employed, not looking for work”, “disabled, unable to work”, and “other” increased significantly from before to after the virtual experience. Table 4 summarizes the results from pre to post, including the change scores, with the greatest increase at post found in the “not employed, looking for work” group (*M* = 0.89, *SD* = 0.79). The findings suggest that wellbeing scores have a positive interaction effect with employment status following participation in Edge of the Present.

#### 3.3.3. Positive and Negative Mood

No significant interactions between the covariables and time were reported.

Three questions were asked of the participants in relation to sense of presence. They included overall enjoyment, strength of presence/being there, and overall comfort level. Sample means were analyzed to determine the average scores of participants in relation to immersion in the virtual experience. Examination of the means indicate that enjoyment levels scored the highest (*M* = 4.58, *SD* = 0.633), followed by overall immersion, “being there” (*M* = 4.29, *SD* = 0.719), and comfort levels (*M* = 4.22, *SD* = 0.811). Scores averaging above four suggest that participants predominantly answered “very good” or “excellent” to the questions.

### 3.4. Open-Ended Comments

The following are a selection of open-ended comments received after participants completed Edge of the Present.

“… I just love coming back here. I have treatment resistant depression and I’ve done traditional therapy as well as the Ketamine trials and all of that. But nothing has made me feel instantly better than this virtual reality work. This is the seventh time that I’ve been back and I just can’t get enough of it. Maybe it’s because I have an addictive personality, but I just feel like my mood is instantly better!”

“I just didn’t know how badly I needed this. It just made me feel instantly relaxed and calm. I’ve been going through a lot today and after exploring the rest of the festival, I just feel like I needed something to make me feel good again. I’m not sure what it was… if it’s getting back to nature? I understand that it’s not real but there’s something about being in nature that makes you feel like you can breathe again.”

“That felt really surreal for me. There is an element of discomfort initially because when you’ve got the headset on, you can’t see your hands. Even though I can open and close things, it was difficult looking down and not seeing my hands in front of me. That’s mainly because I do a lot of yoga and I’m very aware of my body and its effect on my surroundings. But once I got over that and came back again, I realised that I really would have benefited from this if I had this as a teenager. When I was 14, I was institutionalized. When all you’re doing is looking at the same walls all day, I would’ve loved something like this to help me escape that environment.”

## 4. Discussion

In this study, we examined the efficacy of virtual reality technology as a potential tool for the cultivation of positive future thinking for those individuals with a lived experience of suicidality and depression. Despite the effectiveness of virtual reality technologies in health and clinical applications, such as anxiety, posttraumatic stress, or obsessive-compulsive disorder, as evidenced by Bouchard and Rizzo’s [18] book, research has yet to investigate the benefits of using mixed reality technology to address components of depression and suicide. 

We found that amongst all participants, Edge of the Present led to significant reductions in hopelessness scores with an even greater reduction found within the 54 participants who sought help for their mental health by an external organization or professional. Given the link between hopelessness and positive future thinking in parasuicide and depression, it is reasonable to assume that the study strongly affected those individuals with already lowered levels of anticipated positive future episodic thinking [14,33]. Similarly, as predicted, positive mood and well-being increased across all participants following the virtual experience, as well as a subsequent decrease in negative mood. These findings further reinforce the power of imagery-based processing over traditional verbal processing methods of emotional material on negative and positive affect. Together, the results support immersive virtual reality technology as a potentially beneficial mental health intervention, offering new prevention and treatment possibilities to traditional approaches. 

Demographic variables were analyzed for their interaction with hopelessness, mood, and well-being using a mixed-model ANOVA. The interaction between hopelessness against the variable’s “employment” and “sought help” demonstrates the impact of the immersive, mixed reality environment on a participant’s current experience of their mental health. As replicated by previous research, our results suggest that hopelessness remains a significant determinant for participants who sought help for their mental health previously, and that when targeted by positive mental imagery-based processing methods, significant decreases may be found amongst this population group. The interplay between employment and hopelessness, as well as employment and well-being, is also worthy of exploration, revealing that regardless of employment status, Edge of the Present provided a space that fostered hopefulness and well-being amongst all groups. In particular, the group that was unemployed but looking for work exhibited the greatest decrease in hopelessness and subsequent increase in well-being. Unemployment and financial loss are associated with a range of health and social problems, including poor well-being, as well as depression and anxiety. According to Pharr et al. [34], individuals with either less or greater than one year of unemployment, reported significantly worse mental health scores than their employed counterparts. Furthermore, those who were unemployed, but not looking for work, had more favorable mental health scores than those who were involuntarily unemployed. Taken into consideration, and given our own results, it is possible to assume that those seeking employment benefited from Edge of the Present, given their elevated levels of stress and uncertainty. These findings suggest the possibility of traditional clinical interventions working collaboratively with immersive, virtual reality technologies to advance the field of mental health and addressing the current shortcomings in the area of future thinking, depression, and suicide. 

Currently, most virtual reality experiences deployed in mental health interventions are limited in their capacity to sustain the sense of “presence” for users—the feeling of being within a virtual environment—particularly as users cannot walk around a virtual environment freely or physically interact with objects [30]. However, the power of virtual reality to create transformative experiences is often a result of high levels of presence [18]. To improve the user’s experience and to support the cultivation of positive imagery, participants within our study were able to interact with the virtual world (i.e., opening a door or window). The embodied engagement—and the responsive nature of the VR environment, triggered by user actions—enabled embodied learning [35], with participants becoming sensitized to the positive effects of engaging with their surroundings. The mixed-reality platform, which allowed users to physically engage with the room whilst visually exploring the virtual world, provided users with a sense of agency in their virtual journey. High levels of presence amid responders, thus, contributed to diminished feelings of hopelessness, as well as an improvement in mood and well-being amongst all participants. 

Within nature-based therapy, considerable evidence suggests the restorative effects of natural scenery and its associated link with lowered negative emotions and improved positive feelings [36]. The Edge of the Present, comprised of seven differing landscapes—from the snow-covered mountains to the tropical rainforest—achieved a high level of “presence” (known as “communicative realism” [18]) as referred to in a VR setting, whereby participants were heavily immersed in a virtual environment, which could change before them with longer engagement times (e.g., snow falling from the ceiling, grass growing at their feet). In line with previous research on nature-based therapy, the experience of a lush green meadow improved mood and wellbeing, and decreased levels of hopelessness across all participants despite the experience being entirely virtual. As stated by a participant of the study in their post-experience interview, “…I get it that it’s not real but there’s something about being in nature that makes you feel like you can breathe again”. A participant in a separate, qualitative study of the platform further clarified that there was an advantage in the rapid onset of the experience, which yielded satisfaction far more quickly that a real-world excursion in nature.

Unlike most VR developed for health intervention purposes, Edge of the Present is distinctive as it was developed by an arts-based research team for public exhibition in an arts festival—as well as for the purpose of developing an intervention tool. The level of aesthetic refinement and attention to the design of an enjoyable, stimulating experience are important—both in enhancing the functionality (i.e., compelling sense of presence) and in positioning the intervention as something one would choose to do for pleasure. It is feasible that, in the future, Edge of the Present could be adapted for use in clinical/care settings or domestic/community environments, preserving this aspect of a leisure activity.

Despite our positive results, the current study had two important limitations. First, the general attendees who attended the Big Anxiety Festival expressed an enthusiasm and willingness to engage in the entirety of the festival, which also included multiple virtual reality works regarding mental health. Participants may have been biased in their responses, as their engagement with other works of the festival may have impacted their responses. Future research will investigate the effectiveness of Edge of the Present outside the context of a festival, to determine its impact on participants in isolation from other virtual works. Second, the demographic composition of the sample was uneven in relation to gender, employment status, and ethnicity. Therefore, the findings may not accurately reflect the general populous. Future research will ensure that each demographic group has even representation across the study.

## 5. Conclusions

In summary, the development of a custom-built, mixed-reality environment can provide a potential platform for supporting the cultivation of positive future thinking in relation to suicidal ideation and depression. Future developments within this area should regard virtual and mixed reality as an accessible, non-medical platform for engaging individuals who reported with lived experiences of distress or mental ill health—potentially for a wide range of users from those with slightly lowered mood to acute presentations. Future research would benefit from inclusion of a detailed, richly textured qualitative component to determine respondents’ immediate reactions to the experience accompanied by follow-up to determine the impact of the experience over time. 

## Figures and Tables

**Table 1:** Demographic variables of entire sample (N = 79).

Baseline Characteristic	Full Sample	
	*n*	%
Gender		
Female	53	67.1
Male	23	29.1
Non-binary	3	3.8
Age		
18–24	18	22.8
25–34	19	24.1
35–44	13	16.5
45–54	13	16.5
55–64	14	17.7
65+	2	2.5
Preferred language		
English	58	73.4
Other	21	26.6
Education		
Secondary school	10	12.7
University/college	69	87.3
Employment		
Employed, full-time (FT)	24	30.4
Employed, part-time (PT)	25	31.6
Not employed, looking for work	5	6.3
Not employed, not looking for work	1	1.3
Retired	4	5.1
Disabled, unable to work	1	1.3
Student	14	17.7
Self-employed	4	5.1
Other	1	1.3
Virtual reality experience		
None at all	4	5.1
A little	11	13.9
Quite a bit	51	64.6
A lot	13	16.5
Sought help		
Yes	54	68.4
No	25	31.6

**Table 2:** Pre and post scores for repeated-measures *t* test on hopelessness, mood, and well-being (N = 79).

Variable	Pre		Post	
	*M*	*SD*	*M*	*SD*
Beck Hopelessness Scale	5.32	4.31	* 3.44	3.12
Positive and Negative Affect Schedule (Positive Mood)	30.71	7.87	* 35.91	6.37
Positive and Negative Affect Schedule (Negative Mood)	16.98	5.75	* 14.13	4.44
Short Warwick-Edinburgh Mental Well-Being Scale	3.36	0.64	* 3.80	0.53

* Mean difference is significant at the 0.05 level (2-tailed).

**Table 3:** Pre-post and change scores for interaction effect of Employment × Hopelessness (N = 79).

Employment × Hopelessness	Pre		Post		Change Score	
	*M*	*SD*	*M*	*SD*	*M*	*SD*
Employed, FT	3.29	2.68	* 2.67	2.04	0.63	2.01
Employed, PT	6.84	4.39	* 4.28	3.29	2.56	3.62
Not employed, looking for work	5.80	5.63	* 3.00	1.58	2.80	4.15
Retired	4.00	3.46	* 3.00	1.63	1.00	3.46
Student	5.14	3.57	* 3.07	2.92	2.07	2.46

* Mean difference is significant at the 0.05 level (2-tailed).

**Table 4:** Pre-post and change scores for interaction effect of employment x well-being (N = 79).

Employment × Well-Being	Pre		Post		Change Score	
	*M*	*SD*	*M*	*SD*	*M*	*SD*
Employed, FT	3.64	0.65	* 4.00	0.57	0.36	0.48
Employed, PT	3.23	0.45	* 3.64	0.41	0.41	0.41
Not employed, looking for work	3.09	0.70	* 3.97	0.40	0.89	0.79
Retired	3.46	0.55	* 3.89	0.65	0.43	0.45
Student	3.38	0.74	* 3.74	0.54	0.37	0.58
Self-employed	3.43	0.68	* 3.50	0.60	0.07	0.08

* Mean difference is significant at the 0.05 level (2-tailed).

## Data Availability

Data available upon request from the corresponding author. The data are not publicly available due to privacy restrictions.
